# Simultaneous portal and hepatic vein embolization before major liver resection

**DOI:** 10.1007/s00423-020-01960-6

**Published:** 2020-08-24

**Authors:** Jan Heil, Erik Schadde

**Affiliations:** 1 Institute of Physiology, University of Zurich, Winterthurerstr. 190, CH-8057 Zurich, Switzerland; 2 Department of General, Visceral and Transplant Surgery, University Hospital Frankfurt, Goethe-University Frankfurt, Frankfurt, Germany; 3 Department of Surgery, Cantonal Hospital Winterthur, Zurich, Switzerland; 4 Department of Surgery, Rush University Medical Center Chicago, Chicago, IL USA

**Keywords:** Liver, Hypertrophy, Future liver remnant, Liver regeneration, Portal vein embolization

## Abstract

**Background:**

Regenerative liver surgery expands the limitations of technical resectability by increasing the future liver remnant (FLR) volume before extended resections in order to avoid posthepatectomy liver failure (PHLF). Portal vein rerouting with ligation of one branch of the portal vein bifurcation (PVL) or embolization (PVE) leads to a moderate liver volume increase over several weeks with a clinical dropout rate of 20–40%, mostly due to tumor progression during the waiting period. Accelerated liver regeneration by the Associating Liver Partition and Portal vein Ligation for Staged hepatectomy (ALPPS) was poised to overcome this limitation by reduction of the waiting time, but failed due increased perioperative complications. Simultaneous portal and hepatic vein embolization (PVE/HVE) is a novel minimal invasive way to induce rapid liver growth without the need of two surgeries.

**Purpose:**

This article summarizes published results of PVE/HVE and analyzes what is known about its efficacy to achieve resection, safety, and the volume changes induced.

**Conclusions:**

PVE/HVE holds promise to induce accelerated liver regeneration in a similar safety profile to PVE. The demonstrated accelerated hypertrophy may increase resectability. Randomized trials will have to compare PVE/HVE and PVE to determine if PVE/HVE is superior to PVE.

## Introduction

Regenerative liver surgery encompasses methods to increase the future liver remnant (FLR) before resection to expand the limitations of technical resectability of liver tumors. In the 1980s, Kinoshita et al. [1] discovered portal vein embolization (PVE) to protect the liver from tumor thrombi arising from hepatocellular carcinoma, and Makuuchi et al. [2] proposed PVE to allow resections in patients with primary liver tumors with small liver remnants. A Memorial Sloan-Kettering analysis of 1803 patients first showed that the number of resected liver segments had a higher impact on complications after liver resection than the complexity of the performed surgery (bile duct reconstruction, etc.) [3]. Beyond its impact on blood loss, the number of resected liver segments was the main predictive variate for morbidity and mortality and yielded a nearly linear association between the number of resected liver segments and complications. Later analyses confirmed that remnant volume is the main determinant of liver resection outcome [4].

With the advent of routine computed tomography (CT) volumetry, the focus shifted from the resected liver mass to the remaining liver volume [5]. The standardized future liver remnant (sFLR) was proposed for a more accurate estimation of the volume requirement after resection. In sFLR, the total liver volume is estimated by biometric data (body surface area or body weight) which excludes confounders as tumor volumes or dilated bile ducts and keeps the denominator stable when growth is assessed over multiple scans [6]. After systematic studies by the MD Anderson group, a minimal required sFLR of 20–30% became the generally accepted cutoff for healthy livers and ≥ 40% in patients with abnormal histology like cirrhosis in order to avoid posthepatectomy liver failure (PHLF) [4, 5, 7, 8]. To obtain volumes beyond this cutoff, PVE is used to increase the remnant volume with a moderate growth over several weeks [9]. A randomized study of patients undergoing PVE vs. no PVE prior to major hepatectomy was only performed in one study [10]. However, no strict volumetric criteria existed for inclusion; patients were included with and without cirrhosis and with mixed tumor types. In this study, only cirrhotic patients showed a reduction in complications with PVE, but patients with normal livers did not profit from routine PVE prior to major hepatectomy. A randomized study of PVE vs. no intervention in patients with a sFLR < 20–30% however appears unethical today due to the risk of PHLF and perioperative death and therefore convincing controlled clinical data are lacking for regenerative liver surgery in general.

In recent years, strategies different from PVE have been proposed to increase the sFLR prior to resection. Since volume enhancement is frequently desired in liver resections in two stages, Aussilhou et al. [11] showed that PVL used in two-staged hepatectomies (TSH) is equivalent to PVE in terms of its effect to make the liver grow. Kishi et al. [12] showed that adding a segment 4 embolization to PVE enhances the effect and developed the concept of high-quality PVE (HQPVE) over the last years. In 2012, the novel “Associating Liver Partition and Portal vein Ligation for Staged hepatectomy” (ALPPS) technique [13] demonstrated that hypertrophy after portal vein occlusion can be as extensive and fast as regeneration after partial hepatectomy itself, if a parenchymal transection is added to the portal vein ligation (PVL) during the first stage of the TSH. [14] In 2016, Guiu et al. [15] presented a novel interventional technique to induce rapid hypertrophy, the liver venous deprivation technique (LVD). In LVD, the hepatic in- and outflow of the right hemiliver are simultaneously occluded by using PVE and Amplatzer Vascular Plugs for the hepatic veins (AVP, Abott Vascular, formerly St. Jude Medical). This new technique increased liver regeneration comparable in scale and speed with ALPPS.

This review summarizes the current knowledge about the new method of simultaneous portal and hepatic vein embolization (PVE/HVE), a kind of “turbo”-PVE, and investigates its potential as yet another recent innovation in regenerative liver surgery.

## Portal vein rerouting and two-stage hepatectomies

Both PVE and PVL allow to resect large or multiple tumors when more than 70% of the liver needs to be removed [16]. The main drawback of these approaches is the relative small effect on liver regeneration resulting in a drop-off rate of 20–30%, mostly due to tumor progression, partially bit also due to failure to grow [9, 17]. While PVE is used either in conjunction with a TSH or not, PVL builds on the concept of TSH. TSH was initially described for multifocal colorectal liver metastasis (CRLM) [18] and later also for neuroendocrine tumor metastases [19]. During a first stage the main tumor mass was resected and then in a second stage, after a median of 4 months (3–7.5), the remaining tumor was resected [18]. The initial intent of the two stages was to reduce the risk of a simultaneous resection of liver metastases in both hemilivers by allowing recovery of the patient and the liver before a second repeat hepatectomy. Only in 6 of 16 cases in this seminal study, the surgeons additionally performed embolization to increase the liver remnant prior to the second stage [18]. In 2003, Kianmanesh et al. [19] demonstrated an entire series of TSHs using PVL during the first stage in every case after cleaning of the FLR (usually the left hemiliver) followed after a median of 6 weeks (4–8) by a right hepatectomy after an adequate hypertrophy was achieved. In contrast to the TSH that Adam et al. [18] had described, the two procedures followed each other rather rapidly, and generally the main tumor mass was resected with the right liver during the second stage [19].

In 2012, Schnitzbauer et al. [13] presented a technically novel type of TSH under the name of “in-situ-split hepatectomy”, which was subsequently baptized as ALPPS in an Editorial by Clavien et al. [20]. In ALPPS, the TSH with PVL was combined with a transection of the liver parenchyma (in-situ-split) to allow resection of borderline resectable liver tumors after a median of only 9 days [13]. The new method increased hypertrophy and found a lot of enthusiastic follower, but was also criticized for its excessively high complication rates in some of its pioneering centers [21, 22]. The novel phenomenon of rapid hypertrophy was welcomed by many since it allowed faster resection and extended the limitation of technical resectability [23]. However, ALPPS remains tied to the concept of performing bilobar resections in two stages. Using ALPPS for tumors like perihilar cholangiocarcinoma (PHCC) or large unilobular tumors conceptually made no sense, since patient with hilar tumors and large masses should really only undergo one operation [21]. ALPPS therefore, as a clinical routine for all situations of borderline resectability, remained a highly controversial and—despite all the hype—marginally used technique [24].

## Simultaneous portal and hepatic vein embolization

Since the first publication of LVD in 2016 by Guiu et al. [15], 7 more original studies have been published about PVE/HVE resulting in 8 series overall. Three of these are case series [15, 25, 26], and 5 are comparative studies (Table 1) [27–31]. In addition, a systematic review was published about portal and hepatic embolization including staged and simultaneous approaches, fairly early given the relative paucity of original data at the time point of its publication [32].

**Table 1:** Study designs and outcomes of embolization procedures after PVE/HVE

Author	Year	Study design	Patients *n*	Type of tumor (only PVE/HVE)	HVE-technique	Embolized hepatic vein	Procedure-related complications *n*	Hospitals stay
Guiu et al. [15]	2016	Case series	PVE/HVE: 7	CRLM: 2 HCC:1 IHCC:3 PHCC:1	Transhepatic AVP + NBCA/lipiodol	RHV: 7	Pain: 5 Fever > 38°: 5	3 days (2–5)
Guiu et al. [25]	2017	Case series	PVE/HVE: 10	CRLM: 7 PHCC: 1 Other: 2	Transhepatic AVP + NBCA/lipiodol	RHV+MHV: 10	Asthenia Grade 2: 6 Grade 3: 2 Pain: 6 Fever > 38°: 3	3 days (2–5)
Le Roy et al. [26]	2017	Case series	PVE/HVE: 7	CRLM: 2 IHCC: 1 PHCC: 2 Other: 2	Transjugular AVP	RHV: 4 MHV: 2 RHV+MHV: 1	0	n.r.
Hocquelet et al. [27]	2018	Comparative study	PVE/HVE: 6 PVE: 6	PHCC: 6	Transjugular AVP	RHV: 6	0	24–48 h
Panaro et al. [28]	2019	Comparative study	PVE/HVE: 13 PVE: 16	CRLM: 10 HCC: 3	Transhepatic AVP + NBCA/lipiodol	RHV: 13	0	n.r.
Kobayashi et al. [29]	2020	Comparative study	PVE/HVE: 21^*^ PVE: 39	CRLM: 10 HCC: 2 PHCC: 8	Transjugular AVP	RHV: 18 RHV+MHV: 2	Hemobilia: 1	n.r.
Le Roy et al. [30]	2020	Comparative study	PVE/HVE: 31 PVE: 41	CRLM: 18 HCC: 5 IHCC: 2 PHCC: 5 Other: 1	Transjugular AVP	RHV: 27 RHV+MHV: 3 MHV: 1	0	1 day
Laurent et al. [31]	2020	Comparative study	PVE/HVE: 37^+^ PVE:36	CRLM:23 IHCC: 7 HCC: 4 NET: 2	Transjugular AVP + NBCA/lipiodol	RHV: 29 RHV+MHV: 8	Dindo-Clavien: I: 34 II: 3	1.4 days (1–5)

*n.r* not reported, *AVP* Amplatzer Vascular Plug, *CRLM* colorectal liver metastasis, *HCC* hepatocellular carcinoma, *IHCC* intrahepatic cholangiocarcinoma, *MHV* middle hepatic vein, *NBCA/lipiodol* N-butyl-cyanoacrylate and iodized oil, *NE*: neuroendocrine tumor, *PHCC* perihilar cholangiocarcinoma, *PVE* portal vein embolization, *PVE/HVE* simultaneous portal and hepatic vein embolization, *RHV* right hepatic vein

^*^Tumor type and information about the embolization were not given in one patient who failed to achieve liver resection

^+^Tumor type of one patient was not given

The number of patients included in all reports ranges between 6 [27] and 37 patients [31], yielding a total of 132 patients who underwent PVE/HVE in the published experience so far [15, 25–31]. Seven of 8 series included both primary and secondary liver tumors [15, 25, 26, 28–31]. The most common tumor type was CRLM (55% of all reported patients). One comparative study reported the experience of PVE/HVE in PHCC alone [27].

### Technical aspects of PVE/HVE

Guiu et al. [15, 25] called his technique a “deprivation of liver veins”, because he did not only place AVPs in the larger hepatic veins, but also filled the smaller contributories with a mixture of N-butyl-cyanoacrylate and iodized oil (NBCA/lipiodol). In LVD, the right portal vein is embolized—without segment 4 embolization—and during the same session, the right hepatic vein is occluded by the use of AVPs followed with the addition liquid embolization with NBCA/lipidol of the small hepatic veins flowing to the AVPs. In a technically impressive and perfectionist approach, even visible venous collaterals that arise even during the procedure are embolized. In the subsequent article, Guiu et al. [25] then demonstrated a modified version of the LVD, the so-called extend liver venous deprivation (eLVD). eLVD provides an additional occlusion of the right middle hepatic vein using AVPs and NBCA/lipiodol. The eLVD technique, a “turbo” version of LVD in terms of hypertrophy was used in 4 further published series in a limited number of cases (Table 1) [26, 29–31]. Impressively, the Montpellier LVD technique is already being tested in an ongoing randomized controlled trail (RCT) of 8 centers in France (NCT03995459). However, the majority of published articles, which are not from the Montpellier group, except one article from Bordeaux, [31] did not use the additive liquid embolization of hepatic veins following AVP occlusion, but only placed AVPs without much attention to the small veins flowing to the AVPs (Table 1) [26, 27, 29, 30]. Also, the Montpellier group used a transhepatic approach for the hepatic vein embolization [15, 25, 28], which has also been adopted by the Bordeaux group [31]. All other groups use the more common transjugular approach as their standard method [26, 27, 29, 30].

Two studies proposed the new name “bi-embolization” to their version of PVE/HVE performed without liquid embolization of smaller contributories [26, 30]. In contrast, the Lausanne group calls their approach “LVD”, while they did not actually use liquid embolization. In certain sense, “LVD” for procedures without liquid embolization is a misnomer [29]. The Bordeaux group in contrast actually used an additional liquid embolization like the LVD technique and should have called their technique “LVD”, but unfortunately created yet another name, “RASPE” (radiological simultaneous portohepatic vein embolization) [31].

**Fig. 1:**
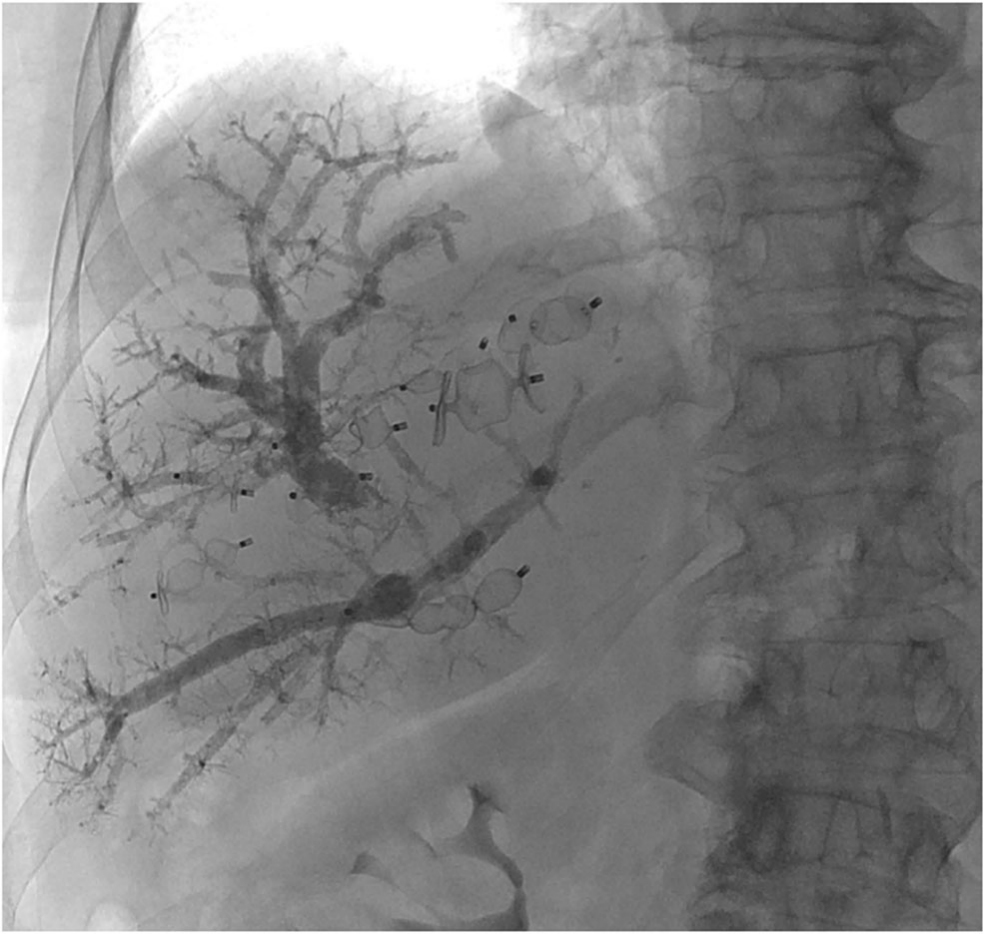
CT scan of a patient using multiple AVPs for right-sided hepatic vein embolization

In a Delphi process leading to the collaborative Dragon trial (DRAGON: NCT04272931), a decision among participating centers was made to refrain from the additional liquid embolization of small contributories due to the perceived risk of liquid embolization of the venous system, the right heart circulation and the lungs. Figure 1 shows a CT scan of a patient using multiple AVPs for right-sided hepatic vein embolization. However, to avoid further confusion, the generic descriptive PVE/HVE was used for the procedure.

### Feasibility of resection after PVE/HVE

Taking all published series into account, 132 patients underwent PVE/HVE to increase the FLR prior to surgery [15, 25–31]. Mean or median time between PVE/HVE and surgery ranges between 21 [27] and 49 days (interquartile range (IQR) 20–210) [26], while one series did not report this time interval at all [30] (Table 2). During that interval, 16 patients developed progression of disease [15, 26, 27, 29–31], and in one patient, liver function growth was insufficient [25]. Overall, 115 of 132 patients (87%) achieved surgical resection after PVE/HVE, resulting in a drop-off rate of 13% [15, 25–31].

**Table 2:** Postoperative outcome of liver resection after PVE/HVE

Author	Patients (PVE/HVE) n	Time between embolization and surgery (days)	Planned/conducted hepatectomy (PVE/HVE) *n*	Feasibility of resection (PVE/HVE) *n* (%)	R0 *n* (%)	Postoperative complications (PVE/HVE) (Dindo-Clavien)	PHLF *n*	Mortality *n*
Guiu et al. [15]	7	23 (range 13–30)	Ext. right HE: 6	6/7 (86%)	5/6 (83%)	Overall: 1 Major: 1 (17%)	0	8-week: 1
Guiu et al. [25]	10	31 (range 22–45)	Right HE: 1 Ext. right HE + S4: 6 Ext. right HE +4 + 1: 2	9/10 (90%)	9/10 (90%)	Overall: 2 Major: 1 (11%)	0^++^	90-day:0
Le Roy et al. [26]	7	49 (IQR 20–210)	Ext. right HE to S4: 4^ϕ^ Ext. right HE: 3^ϕ^	6/7 (86%)	6/6 (100%)	Overall: 4 Major: 2 (33%)	0^++^	90-day:1
Hocquelet et al. [27]	6	21	Ext. right HE + S1: 3^*^ Ext. right HE + S1 + S4a: 6	4/6 (67%)	3/6 (50%)	n.r.	2^++^	90-day:0
Panaro et al. [28]	13	38	Right HE: 13	13/13 (100%)	n.r.	Overall:10 Major (≥IIIa): 1 (8%)	3^#^	0^&^
Kobayashi et al. [29]	21^*^	35 (IQR 23–109)	Right HE: 9 Ext. right HE: 11	20/21 (95%)	n.r.	Overall: 11 Major (>III): 7 (35%)	n.r.	0^&^
Le Roy et al. [30]	31	n.r.	Right HE: 8 right HE + S1: 1 right HE + S4: 5 right HE + S1 + S4: 9 ALPPS: 2^##^	25/31 (81%)	n.r.	Overall: 15 Major (>IIIa): 5 (20%)	n.r.	90-day:3
Laurent et al. [31]	37^+^	36 (range 16–47)	ext. right HE: 22 right HE: 10	32/37(86%)	31/32 (97%)	Overall: 32 Major (≥ IIIa): 6 (19%)	0	n.r.

*n.r.* not reported *ALPPS* associating liver partition and portal vein ligation for staged hepatectomy *ext. right HE*: extended right hepatectomy *right HE*: right hepatectomy *IQR:* interquartile range *PHLF* post-hepatectomy liver failure *R0* microscopically clear margin *S1* liver segment 1 *S4* liver segment 4 *S4a* liver segment 4a

^&^Information was not given if mortality referred to 30- or 90-day mortality

^ϕ^No differentiation between planned and conducted surgery

^*^Procedures only given for the entire cohort (PVE/HVE and PVE)

^++^PHLF according to the 50–50 criteria

^#^PHLF according to the ISGLS criteria

^##^Reason for performing ALPPS was unclear in the PVE/HVE group

In the studies where PVE/HVE was compared with PVE, surgery was performed between a mean or median of 21 [27] to 45 days (standard deviation (SD) ± 5) [31] after PVE, showing no difference between PVE/HVE and PVE (Table 4) [27–29, 31]. Again, one series did not report on that interval [30]. For PVE, resectability ranges between 76 [30] and 94% [28]. In two comparative series, the feasibility of resection after PVE/HVE vs. PVE was not specifically analyzed due to the small study size [27, 28]. However, in the remaining 3 comparative series, no difference was seen in resectability between both approaches [29–31].

The importance of the endpoint feasibility of resection to assess methods of regenerative liver surgery was recently demonstrated by the Scandinavian LIGRO trial (NCT02215577)—the first RCT of ALPPS vs. TSH [33]. Patients with borderline resectable CRLM were randomized either to ALPPS or TSH with a PVL during the first stage or a PVE between the stages. Patients included in this trial were highly selected and received induction chemotherapy. Both approaches, ALPPS and TSH demonstrated a relatively high morbidity (43% major complications (≥ IIIa) in both procedures) and high mortality (ALPPS: 9.1% vs. TSH: 10.7%). However, patients who underwent ALPPS had a 92% vs. 57% (TSH) resectability (*p* < 0.001). While in the ALPPS cohort patients underwent both stages within a mean of 11 days (SD ± 11), in the TSH cohort the second stage was performed after a mean of 43 days (SD ± 15) due to the slower hypertrophy (*p* < 0.001). During that time, 16% in the TSH cohort had tumor progression, and 27% demonstrated insufficient liver growth. Interestingly, in a follow-up evaluation [34], the ALPPS cohort also demonstrated an improved median survival of 46 months compared with 26 months after TSH (*p* = 0.028). For the first time, the LIGRO trial demonstrated an effect of a surgical resection technique on survival in metastases surgery based on randomized data. It appears as if rapid resection of the entire tumor load in CRLM matters.

### Safety of PVE/HVE

The interventional radiology procedure PVE/HVE was successfully performed in all 132 patients in the published series, and no severe adverse events were reported (Table 1) [15, 25–31]. No difference of complications after the intervention itself was reported in the 5 comparative series between PVE/HVE and PVE [27–31]. Theoretical concerns about liver necrosis due to the simultaneous occlusion of the hepatic in- and outflow were not observed in these initial clinical reports [15]. As far as the effect of PVE/HVE on the liver is concerned, 8 days after LVD the transaminases remain slightly elevated, but there was no sign of liver necrosis in histology, which was confirmed by two further studies [28, 31]. It has to be assumed that the devascularized lobe remains viable by arterial blood flow alone [35]. It has to be postulated that new venous outflow collaterals enable the drainage of the arterial blood [36]. In any case, arterial blood flow appears sufficient to avoid liver necrosis at a larger scale.

### Postoperative outcome

The majority of patients who underwent PVE/HVE as preparation later underwent a major hepatectomy (Table 2) [15, 25–30]. Seven of 8 series reported on the postoperative complications of this hepatectomy using the Dindo-Clavien classification [15, 25, 26, 28–31], while one comparative study did not provide information about the postoperative outcome according to the Dindo-Clavien classification [27]. In these 7 series, 111 patients underwent surgery after PVE/HVE [15, 25, 26, 28–31]. Overall complications occurred in 75 of those (68%), while complications at least III were reported in 23 patients (21%). PHLF occurred in 5 patients (5%) [27, 28]. In one series, 50–50 criteria were used of assessment [27], while the other studies defined PHLF according to the International Study Group of Liver Surgery (ISGLS) criteria [28].

All series except one [31] reported their mortality [15, 25–30], but two series did not mention if perioperative mortality rate referred to the 30- or 90-day mortality [28, 29]. Of the 111 patients who underwent surgery after PVE/HVE, 5 patients died in the postoperative course, resulting in a mortality rate of 5% [15, 26, 30]. One series reported that one of 6 patients died 10 days after surgery due to postoperative pneumonia [15]. In another study, one patient died 10 days after coiling of the common hepatic artery for postoperative hemorrhage [26]. No further information was provided on the death of 3 more patient within 90 days after resection [30].

When morbidity and mortality of PVE/HVE were compared with PVE in the comparative studies, no difference was observed between the two (Tables 2 and 4) [27–31]. However, one series demonstrated a difference in the occurrence of PHLF between PVE/HVE (0%) and PVE (23%) (*p* = 0.012) [31].

Interestingly, all of these mortalities after both ALPPS and TSH were PHLF-related in the LIGRO trial [33]. Overall, 5 patients in all studies available so far developed PHLF following PVE/HVE (Table 2), but none of these cases resulted in a perioperative death [15, 26, 30].

### Volumetric effect of PVE/HVE

All series report on the volume increase of the FLR after PVE/HVE (Table 3) [15, 25–31], but a comparison between the series is difficult due to the inhomogeneity of metrics used to measure liver volume and growth. A standardization should be considered obligatory for future studies.

**Table 3:** Volumetric data of PVE/HVE in all published series

Author	FLR (preintervention) PVE/HVE	Time between embolization and imaging (days)	FLR (postintervention) PVE/HVE	Percent hypertrophy	Degree of hypertrophy	KGR
Guiu et al. [15]	28.2% FLR (range 22.4–33.3)	23 (range 13–30)	40.9% FLR (range 33.6–59.3)	n.r.	12.7% FLR	4.2% sFLR/week
Guiu et al. [25]	20.8% sFLR (SD ±5.1)	7 14 21	31.8% sFLR (SD ± 8.2) 33.4% sFLR (SD ± 7.2) 33.4% sFLR (SD ± 6.7)	53.4%^ε^ 62.5%^ε^ 63.3%^ε^	n.r.	7.6 cc/day (SD ± 2.4)^#^ 0.9 cc/day (SD ± 0.9)^#^ 0.1 cc/day (SD ± 1.3)^#^
Le Roy et al. [26]	21% FLR (IQR 14–37)	22 (IQR 19–28)	30% FLR (IQR 25–47)	52.6% (absolute FLR) (IQR18–188)	n.r.	unclear
Hocquelet et al. [27]	30.5% FLR (IQR 23–35.5)	23.5 (IQR 15–29)	42.3% FLR (IQR 34–47) /58% sFLR (IQR 54–71)	67% FLR (IQR 29–123)	n.r.	n.r.
Panaro et al. [28]	31.2% FLR (SD ±6.5)	21	40.8% FLR (SD ± 7.9%)	n.r.	n.r.	16 cc/day (SD ±7)
Kobayashi et al. [29]	25% sFLR (IQR 23–31)	22 (IQR 17–30)	36% sFLR (IQR 31–40)	35% FLR (IQR 23–54)	8.9% FLR (IQR 6.7–12.8)	2.9% FLR/week (IQR 1.9–4.3)
Le Roy et al. [30]	394 cc (CI 262–478)	26	527 cc (CI 416–662)	51.2% (SD ± 41.7)	10% (SD ± 6)	unclear
Laurent et al. [31]	22.91% FLR (range 16.55–32.15)	31 (± 2)	39.89% FLR (range 30.64–52.92)	61.18% FLR (range 18–201)	n.r.	n.r.

*n.r.* not reported *cc* cubic centimeter *CI* confidence interval *FLR* future liver remnant *IQR* interquartile range *KGR* kinetic growth rate *SD* standard deviation *sFLR* standardized future liver remnant

^ε^Percent hypertrophy (absolute FLR volume) from baseline

^#^KGR (in cc per day) from baseline

Standardized volumetric data by the use of biometric formulas for the sFLR are only provided by two studies [25, 29]. In the series comparing PVE/HVE with PVE, no significant difference was achieved in the sFLR after the respective interventions (Tables 3 and 4) [29]. However, the achieved percent hypertrophy was significantly different of the FLR (35% (IQR 23–54) vs. 24% (IQR 7–40) resp. *p* = 0.03). The same was shown by the comparative study from Clermont-Ferrand group [30], which unfortunately used the metrics “absolute FLR volume” in cubic centimeters (cc). While the liver volume did not differ after PVE/HVE and PVE, the percent hypertrophy (in cc) shows a significant difference: 51.2% (SD ± 41.7) vs. 31.9% (SD ± 34) (*p* = 0.018). The percent hypertrophy also differed between PVE/HVE and PVE in a recently published comparative series, but the starting FLR in the PVE control calls into question, if a regenerative maneuver was indicated in these patients at all [31].

**Table 4:** Outcome after PVE in the comparative series

Author	Patients *n*	FLR (preintervention) PVE/HVE	Time between embolization and imaging (days)	FLR (postintervention) PVE	Percent hypertrophy/degree of hypertropy	KGR	Time between embolization and surgery (days)	Feasibility of resection	Postoperative complications (Dindo-Clavien)
Hocquelet et al. [27]	6	31% FLR (IQR 24–33)	23.5 (IQR 15–29)	39% FLR (IQR 36–42) 37% sFLR (IQR 30–44)	31.3% FLR (IQR 12–40) /n.r.	n.r.	21	5 (83%)	n.r.
Panaro et al. [28]	16	n.r.	21	n.r.	n.r./n.r.	4.8 cc/day (SD ± 4)	37	15 (94%)	Overall: *n* = 7 Major (≥ IIIa): *n* = 3 (20%)
Kobayashi et al. [29]	39	24% sFLR (IQR 20–30)	26 (IQR 20–33)	31% sFLR (IQR 25–38)	24% FLR (IQR 7–40) / 6% FLR (IQR 1.9–9.2)	1.4%/week (IQR 0.7–2.1)	35 (IQR 20–181)	30 (77%)	Overall: *n* = 17 Major (>III): *n* = 11 (37%)
Le Royet al. [30]	41	348 cc (CI 266–547)	27	487 cc (CI 327–612)	31.9% (SD ±34) / 7.5% (SD ±5)	Unclear	n.r.	31 (76%)	Overall: 12 Major (>IIIa): n = 3 (10%)
Laurent et al. [31]	36	31.03% FLR (range 18.33–38.95)	31 (±2)	40% FLR (range 24.11–53.86)	28.98% FLR (range 9.31–61.23) /n.r.	n.r.	45 (±5)	32 (89%)	Overall: 32 Major (>IIIa): *n* = 10 (31%)

*n.r.* not reported *cc* cubic centimeter *CI* confidence interval *FLR* future liver remnant *IQR* interquartile range *KGR* kinetic growth rate *SD* standard deviation *sFLR* standardized future liver remnant

To compare the novel PVE/HVE with PVE, the choice of the PVE control group really matters. The MD Anderson group has shown that PVE can be performed as low- or high-quality procedure [12]. In HQPVE, embolization segment 4 is added to right hemiliver embolization. Any new version of PVE like PVE/HVE has to be compared with HQPVE. In HQPVE, an increase of the sFLR of 20.1% (range: 9.5–57.8) to 29.4% (range: 20.1–75.1) within a median of 30 days should be considered to be the gold standard [37]. The same applies to the growth metrics “degree of hypertrophy (DH)”, which is defined as the sFLR after the intervention minus the sFLR prior to the invention. The DH of 8.9% (IQR 6.7–12.8) for PVE/HVE demonstrated by the Lausanne group [29] or the DH of 12.7% by the Montpellier group [15] have to be compared with 10.1% (0.1–39.9) after HQPVE as published by the MD Anderson group [37]. Unfortunately, the comparison is limited because the Lausanne [29] and Montpellier group [15] preferred to use FLRs instead of sFLR.

To estimate the speed of liver growth, the metrics “kinetic growth rate” (KGR) is routinely used, which is defined as the ratio between the DH and the elapsed time in weeks between embolization and first cross-sectional volumetry in sFLR/week [37]. Unfortunately, 2 series did not give any information about the KGR [27, 31], and in 2 further series, the calculation of the KGR is unclear [26, 30]. Two used percent of total liver volume increase per week as their metrics for kinetic growth (Table 3) [25, 28].

While the initial study from Montpellier reported a KGR of 4.2% sFLR/week for PVE/HVE after a median of 23 days (range: 13–30) [15], the MD Anderson group gave a KGR of 2.4% sFLR/week (range: 0.2–9.4) after a median of 30 days (range: 14–54) for PVE. Of note, 63% of patients underwent HQPVE in this study. Although the KGR after PVE/HVE was two times higher in the Montpellier group than that of HQPVE, it should be noted that the volumetric assessment was performed 7 days later at the MD Anderson [37], which may introduce a bias towards a higher KGR. Also, none of the patients reported from Montpellier had diabetes or underwent chemo before embolization, [15] while the MD Anderson report included patients with diabetes (6.8%) and chemotherapy (92.3%), which is known to reduce the KGR [7, 37, 38]. The Lausanne group demonstrates a KGR of 2.9% FLR/week (IQR 1.9–4.3) after a median of 22 days (IQR 17–30), while 50% of patients underwent chemotherapy [29].

The report by the Montpellier group about eLVD in 2017 reports a successive volume increase over 3 time points (7, 14, and 21 days) in 3 patients [25]. From day 0 to day 7, the FLR increased from a mean of 20.8% (SD ± 5.1) to a mean of 31.8% (SD ± 8.2) (53% hypertrophy from baseline). From day 7 to day 14, the FLR increased less dramatically from 31.8 to 33.4% (SD ± 7) (63% hypertrophy from baseline) and from day 14 to 21 from 33.4 to 33.4% (SD ± 6.7) (63% hypertrophy from baseline). The Scandinavian LIGRO trial also reports volume data after 7 days [33]. The percent hypertrophy in ml in patients suffering from CRLM was 68% (± 38) for ALPPS and 43% (± 30) for PVE or PVL. Looking at these data, PVE/HVE seems to be right in the middle between PVE and ALPPS [25].

### Function data

There is evidence that ALPPS leads to an incongruent increase in volume and function using technetium-99 m hepatobiliary scintigraphy (^99m^Tc-mebrofenin HBS) [39, 40]. PVE/HVE has also been evaluated for functional changes using ^99m^Tc-mebrofenin HBS in 10 patients [25]. In 3 of those, who underwent eLVD, a serial measurement was performed at days 7, 14, and 21 (according to the volume assessment). Although, these data are based on a small patient sample size, a parallel increase was described in volume and function with a maximal function already at day 7 (65.7% (SD ± 16)). Afterwards, at day 14 and 21, the function does not demonstrate a further increase from baseline (14 days: 57% (SD ± 18) and 21 days: 57% (± 18).

### Limitations

Overall studies about PVE/HVE are characterized by a small study size and highly selected patients. Patients with diabetes for example have been underrepresented [30, 37]. Not more than 5 patients had liver cirrhosis, and only 6 patients had elevated bilirubin before embolization and needed a biliary drainage [27, 28, 30]. In 5 series, patients (*n* = 50) were reported to have received chemotherapy before embolization, but not all series provide information about the specific drugs used [25, 26, 28, 29, 31].

The question if PVE/HVE has long-lasting advantages over PVE can likely not be answered by cohort studies, but requires a RCT. Currently two studies are registered (International DRAGON trial: NCT04272931, LVD France trial: NCT03995459).

## Conclusions

Data from cohort studies demonstrate that PVE/HVE does not result in a higher rate of morbidity and mortality than PVE. It appears that induction of accelerated and more extensive hypertrophy increases resectability compared with PVE. Future RCTs will be able to determine, if PVE/HVE represents a true improvement over PVE.

## Abbreviations

^99m^Tc-mebrofenin HBS: Technetium-99 m hepatobiliary scintigraphy
ALPPS: Associating liver partition and portal vein ligation for staged hepatectomy
AVP: Amplatzer vascular plug
cc: Cubic centimeter
CRLM: Colorectal liver metastasis
CT: Computed tomography
DH: Degree of hypertrophy
eLVD: extended liver venous deprivation
FLR: Future liver remnant
HVE: Hepatic vein embolization
HQPVE: High-quality portal vein embolization
IHCC: Intrahepatic cholonagiocarcinoma
ISGLS: International Study Group of Liver Surgery
KGR: Kinetic growth rate
LVD: Liver venous deprivation
NBCA/lipiodol: N-Butyl-cyanoacrylate and iodized oil
NET: Neuroendocrine tumor
PHCC: Perihilar cholangiocarcinoma
PHLF: Posthepatectomy liver failure
PVE: Portal vein embolization
PVL: Portal vein ligation
RCT: Randomized controlled trial
SD: Standard deviation
sFLR: standardized future liver remnant
TSH: Two-staged hepatectomy
